# What Is Similar, What Is Different? Characterization of Mitoferrin-like Proteins from *Arabidopsis thaliana* and *Cucumis sativus*

**DOI:** 10.3390/ijms26157103

**Published:** 2025-07-23

**Authors:** Karolina Małas, Ludmiła Polechońska, Katarzyna Kabała

**Affiliations:** 1 Department of Plant Molecular Physiology, Faculty of Biological Sciences, University of Wrocław, Kanonia 6/8, 50-328 Wroclaw, Poland; karolina.malas2@uwr.edu.pl; 2 Department of Ecology, Biogeochemistry and Environmental Protection, Faculty of Biological Sciences, University of Wrocław, Kanonia 6/8, 50-328 Wroclaw, Poland; ludmila.polechonska@uwr.edu.pl

**Keywords:** chloroplasts, iron homeostasis, MCF, MIT proteins, mitoferrin-like proteins

## Abstract

Chloroplasts, as the organelles primarily responsible for photosynthesis, require a substantial supply of iron ions. Conversely, due to Fe toxicity, the homeostasis of these ions is subject to tight regulation. Permease in chloroplast 1 (PIC1) has been identified as the primary iron importer into chloroplasts. However, previous studies suggested the existence of a distinct pathway for Fe transfer to chloroplasts, likely involving mitoferrin-like 1 (MFL1) protein. In this work, Arabidopsis MFL1 (AtMFL1) and its cucumber homolog (CsMFL1) were characterized using, among others, Arabidopsis protoplasts as well as both yeast and Arabidopsis mutants. Localization of both proteins in chloroplasts has been shown to be mediated via an N-terminal transit peptide. At the gene level, *MFL1* expression profiles differed between the model plant and the crop plant under varying Fe availability. The expression of other genes involved in chloroplast Fe homeostasis, including iron acquisition, trafficking, and storage, was affected to some extent in both *AtMFL1* knockout and overexpressing plants. Moreover, root growth and photosynthetic parameters changed unfavorably in the mutant lines. The obtained results imply that AtMFL1 and CsMFL1, as putative chloroplast iron transporters, play a role in both iron management and the proper functioning of the plant.

## 1. Introduction

Chloroplasts are mainly the site of photosynthesis; therefore, they play a key role in the primary and secondary metabolism of plants [1]. The proper functioning of this process requires iron ions, which are an important element of photosystems II and I, the cytochrome b6f complex, and ferredoxin. Iron is also necessary for the biogenesis of cofactors such as heme and iron–sulfur (Fe-S) clusters [2].

Chloroplasts contain 80 to even 90% of the total Fe content in leaves and are therefore the compartment richest in this trace element [3]. Iron deficiency negatively affects chlorophyll biosynthesis, results in leaf chlorosis, and leads to remodeling of the photosynthetic apparatus [4]. On the other hand, an excess of this metal due to the production of reactive oxygen species (ROS) is also harmful to plants, especially since the photosynthetic electron transport chain generates free radicals [5]. For this reason, the processes of iron import and export, its storage, and binding to other molecules are strictly regulated in these organelles. Consequently, the iron status of the plant and its response to iron deficiency or excess are controlled, among others, via retrograde signaling from chloroplasts [6].

Iron is transferred from the cytoplasm through the outer chloroplast membrane probably in the form of a Fe^3+^ complex with a chelating compound, and the preferred form seems to be Fe^3+^–citrate, as demonstrated in *Beta vulgaris* and *Brassica napus* [7,8,9]. In the next step, iron is transported through the inner chloroplast membrane to the stroma.

Proteins responsible for iron transport from the cytoplasm to the intermembrane space of chloroplasts have not yet been identified. It is believed that they form a β-barrel structure and belong to a group called OEPs (outer envelope proteins), similar to bacterial porins and channels [10]. The first identified iron importer, located in the inner chloroplast membrane, was the Arabidopsis protein AtPIC1 (permease in chloroplast 1) [11]. A homologous protein, NtPIC1, with a similar function was subsequently demonstrated in *Nicotiana tabacum* [12]. Analyses of *pic1* knockout mutants, as well as the *fro7* mutant characterized by lowered activity of the inner envelope reductase (AtFRO7), responsible for Fe reduction in the intermembrane space, suggest the functioning of an alternative iron transport pathway to chloroplasts, probably with lower substrate affinity [11,13]. One potential mechanism involves the MFL1 (mitoferrin-like 1) protein identified in *A. thaliana*, belonging to the Mitochondrial Carrier Family (MCF) [14].

All yeast and animal and most plant MCF proteins are located in the mitochondria. They are transporters characterized by a conserved structure generally consisting of six transmembrane domains arranged in three repeating modules of about 100 amino acids in length, connected by three short α-helices on the matrix side [15]. Additionally, each protein possesses a characteristic MCF motif [16], and its putative substrate specificity can be predicted based on phylogenetic analysis [16,17], because each subgroup of MCF has specific substrate contact points [18]. Based on this classification, one of the subgroups includes phosphate and iron transporters [17,19], with mitoferrin proteins being representatives of the latter.

Mitoferrins were initially characterized in yeast and animals as mitochondrial proteins; however, homologous sequences encoding both mitochondrial and chloroplast proteins have also been identified in plant genomes [17]. Plant mitoferrins, called MITs (mitochondrial iron transporters), are the least studied group of all mitoferrins found in the three kingdoms of eukaryotes. They have been functionally characterized so far in *Oryza sativa* (OsMIT) [20], *A. thaliana* (AtMIT1 and AtMIT2) [21], *Solanum tuberosum* (StMIT) [22], and *Cucumis sativus* (CsMIT1 and CsMIT2) [23].

Arabidopsis AtMFL1, classified as a plastid protein based on proteomic studies, was identified as a homolog of *Danio rerio* Mitoferrin-2 [14]. It was suggested that chloroplast MCF proteins may be the result of mitochondrial gene duplication and subsequent association of its function with plastids [24] or the effect of plastid targeting of proteins previously functioning as mitochondrial [25,26,27]. Analysis of *atmfl1-1* and *atmfl1-2* knockout mutants showed that this protein is not essential for plants, although its dysfunction results in reduced growth and lowered Fe accumulation. *AtMFL1* expression appears to be iron-dependent and related to the expression of genes encoding proteins involved in chloroplast metabolism [14].

Studies using Arabidopsis are the only ones confirming that *MFL1* (sequence available in the NCBI database under accession number At5g42130) encodes a functional protein. For this reason, we focused on a more comprehensive characterization of the MFL proteins and an explanation of its function in Arabidopsis, as well as extending this characterization to other plant species. For this purpose, we identified the sequence encoding a homologous protein in the genome of cucumber, which, unlike Arabidopsis, is an agriculturally important plant. Based on the available data, we hypothesized that MFL1 proteins localize specifically to chloroplasts within plant cells, and this subcellular targeting is mediated by a distinct N-terminal sequence. This was verified using *A. thaliana* protoplasts. In addition, we assumed that the expression of both genes encoding a putative iron importer could depend on different iron availability and plant species. For this reason, we analyzed the expression levels of *AtMFL1* and *CsMFL1* under both short and long iron-deficiency and -excess conditions. Finally, to confirm whether MFL1 proteins are involved in iron transport and homeostasis in plants, we performed complementation assays in the yeast *Saccharomyces cerevisiae*, as well as functional characterization using *AtMFL1* knockout mutant and overexpression lines.

## 2. Results

### 2.1. Identification of CsMFL1 in Cucumis sativus Genome and Characteristic Features of Plant MFL Proteins

**Figure 1:**
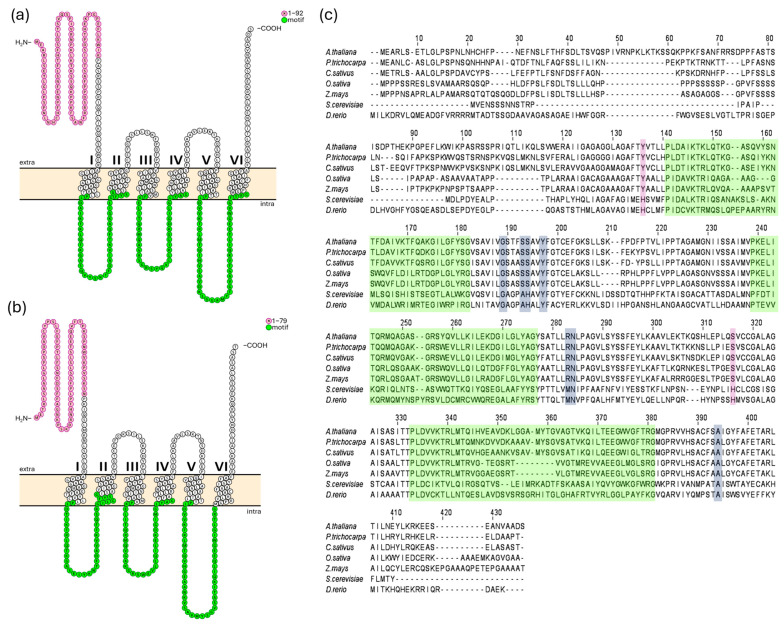
In silico analysis of MFL1 proteins from *A. thaliana* and *C. sativus.* Membrane topology of AtMFL1 (**a**) and CsMFL1 (**b**). Analysis was performed in the CCTOP program (v1.1.0) and visualization in the Protter program (v1.0). The characteristic motif of MCF proteins is marked in green, while the potential chloroplast targeting sequence is marked in pink. Other amino acids in the sequence are marked in white. Extra—intermembrane space, intra—stroma. Alignment of MFL protein sequences (**c**). Sequences of MFL1 proteins from *Arabidopsis thaliana*, *Cucumis sativus*, *Populus trichocarpa*, *Oryza sativa*, and *Zea mays,* as well as mitoferrin 2 from *Danio rerio* and Mitochondrial Iron Transporter from *Saccharomyces cerevisiae* (ScMRS3), were used, and alignment was generated using Clustal O. Green highlights represent the characteristic sequence motif of MCF proteins. Putative substrate contact sites, identified based on animal mitoferrin from *Danio rerio*, are highlighted in gray. Amino acids corresponding to iron-binding sites, shown in mitochondrial iron transporters of *Saccharomyces cerevisiae,* are highlighted in pink.

AtMFL1 protein was first characterized by Tarantino et al. [14]. In this study, its coding sequence was used to discern the full putative genomic sequence of *CsMFL1*. The whole contigs of Borszczagowski cultivar genome were screened by the BLAST^®^ (blastn) program (NCBI server, https://blast.ncbi.nlm.nih.gov/Blast.cgi, 17 January 2017), with further analysis performed using FGENESH and FGENESH+ software (Softberry, Mount Kisco, NY, USA, www.softberry.com, accessed on 17 January 2017). The cucumber *CsMFL1* sequence was identified in ACYN01002741.1 contig and, similar to its Arabidopsis homolog, it contains only 1 exon encoding a putative protein of 391 amino acids in length, with a molecular mass calculated at 41.91 kDa. CsMFL1 shares about 67% sequence homology with the 412-amino acid (44.4 kDa) AtMFL1 protein. Both proteins are predicted to localize to chloroplasts (ChloroP, Localizer), probably in the inner envelope, and have six transmembrane helices, with both ends located in the chloroplast intermembrane space (Figure 1a,b). Additionally, an N-terminal chloroplast transit peptide (cTP) of 92 amino acids in length was identified in AtMFL1 by ChloroP (Figure 1a), a sequence shorter by 6 amino acids than the one identified by Tarantino et al. [14]. Based on protein sequence alignment, a 79-amino acid cTP was determined in CsMFL1, as ChloroP was unable to predict it (Figure 1b). Similar to other proteins belonging to the Mitochondrial Carrier Family, both transporters exhibit its characteristic sequence motif (Figure 1a–c) [16]. On the other hand, in contrast to MIT proteins, the putative conservative iron-binding motif GXXXAHXXY, MN, A (Figure 1c) [18] is altered and consists of GXXXSSXXY, RN, A. Moreover, histidine residues identified as crucial for iron binding in yeast MRS proteins [28] are not present in MFLs (Figure 1c).

### 2.2. Expression Analysis of AtMFL1 and CsMFL1

**Figure 2:**
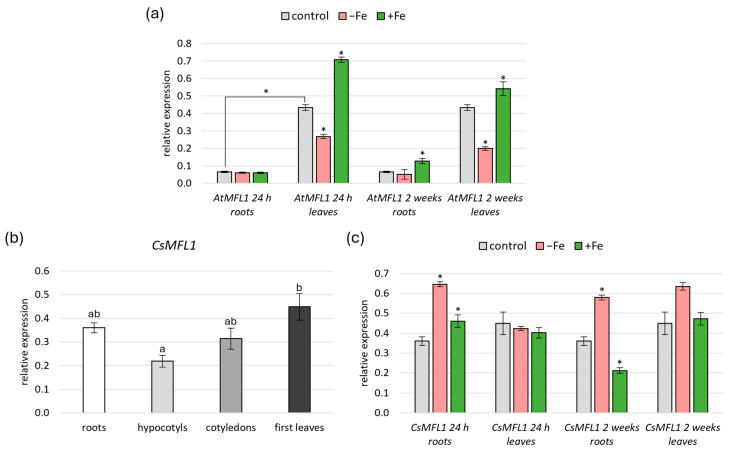
Gene expression analysis of *AtMFL1* and *CsMFL1.* Expression of *AtMFL1* in roots and rosette leaves of 8-week-old plants (**a**) under various iron availability conditions: control, Fe deficiency (−Fe; −FeSO_4_–EDTA +0.05 mM BPS), and Fe excess (+Fe; +0.2 mM FeSO_4_–EDTA) with short-term (24 h) and long-term (2 weeks) treatment. Expression of *CsMFL1* in roots, hypocotyls, cotyledons, and leaves of 2-week-old plants under control conditions (**b**). Expression of *CsMFL1* in roots and leaves of 2-week-old plants under various iron availability conditions (**c**): control, Fe deficiency (−Fe; −FeSO_4_–EDTA +0.03 mM BPS), and Fe excess (+Fe; +1 mM FeSO_4_–EDTA) with short-term (24 h) and long-term (2 weeks) treatment. The expression levels were calculated according to the ΔΔCT method relative to the reference genes *AtCACS* (*Arabidopsis thaliana*) or *CsCACS* (*Cucumis sativus*). The presented values are the means of three biological replicates. Error bars represent standard error (±SE). If not indicated otherwise, asterisks represent statistically significant differences between control and −Fe or +Fe (*p* < 0.05; Student’s *t*-test). Different letters represent statistically significant differences (*p* < 0.05; ANOVA with Tukey’s correction).

A previous study by Tarantino et al. [14] showed that *AtMFL1* is expressed in leaves, with an increased level under constant high Fe supply. The aim of this study was to compare the expression of both *AtMFL1* and *CsMFL1* in all vegetative organs under short- (24 h) and long-term (2 weeks) variable Fe availability (Figure 2a–c). Under control conditions, *AtMFL1* expression was significantly higher in leaves than in roots of 8-week-old plants (Figure 2a). In contrast, *CsMFL1* was expressed at a similar level in roots, hypocotyls, cotyledons, and the first leaves of 2-week-old cucumber plants (Figure 2b), with the only statistically significant difference found between hypocotyls and leaves, but interestingly, not between roots and leaves (Figure 2b). Moreover, significant differences were found between *AtMFL1* and *CsMFL1* expression under iron-deficiency and iron-excess conditions. *AtMFL1* transcript levels were downregulated in leaves under Fe deficiency, with a more significant effect observed during long-term than short-term treatments, and contrarily, they were upregulated under Fe excess, with a smaller effect found under long-term than short-term exposure. In roots, only a slight upregulation of *AtMFL1* was noted after 2-week exposure to Fe excess (Figure 2a). Other trends were observed for *CsMFL1*. In leaves, its expression was not dependent on iron availability under the studied conditions. In contrast, it was upregulated in roots with iron deficiency as well as short-term Fe excess. However, under long-term iron-excess conditions, a decrease in *CsMFL1* transcript level was observed (Figure 2c).

### 2.3. Effect of a Loss of N-Terminal cTP on MFL Localization

**Figure 3:**
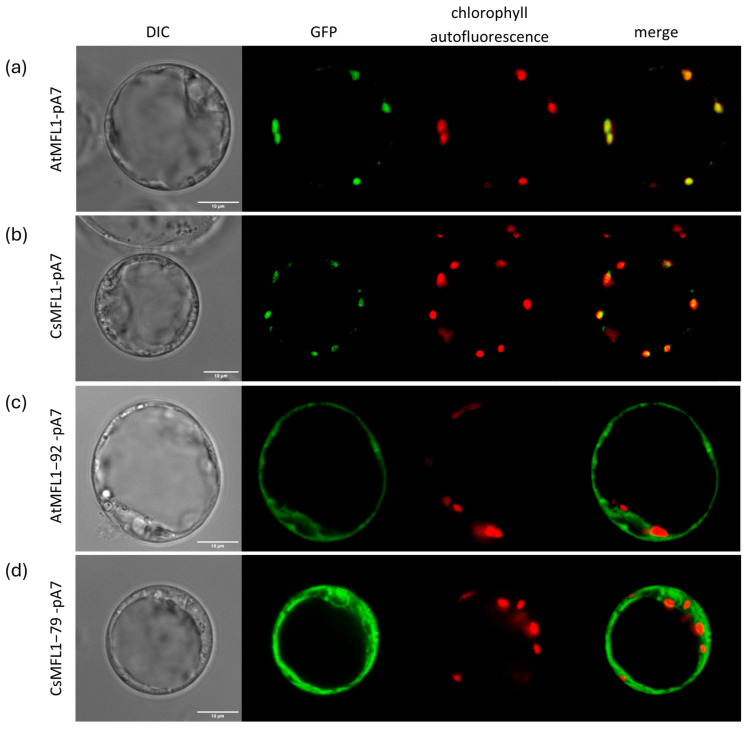
Localization of AtMFL1 and CsMFL1 in *A. thaliana* protoplasts. Subcellular localization of AtMFL1 (AtMFL1-pA7) (**a**); CsMFL1 (CsMFL1-pA7) (**b**); AtMFL1 without N-terminal chloroplast targeting peptide (AtMFL1−92-pA7) (**c**); CsMFL1 without N-terminal chloroplast targeting peptide (CsMFL1−79-pA7) (**d**). Green—GFP fluorescence, red—autofluorescence of chlorophyll, merge—combination of green and red signals. The scale bar corresponds to 10 μm.

In this study, we determined the subcellular localization of both Arabidopsis and cucumber MFL1 proteins in *A. thaliana* protoplasts. Protoplasts were transformed with AtMFL1-pA7 (Figure 3a) or CsMFL1-pA7 (Figure 3b) constructs containing whole protein sequences. As shown in Figure 3, the signals from C-terminally GFP-tagged MFL proteins colocalized with the autofluorescence of chlorophyll, confirming chloroplast localization of both transporters. Moreover, vectors carrying N-terminally truncated proteins lacking putative cTP sequences, AtMFL1−92-pA7 (Figure 3c) and CsMFL1−79-pA7 (Figure 3d), were also introduced into *A. thaliana* protoplasts. In this case, the signals from C-terminally GFP-tagged MFL proteins, observed under a confocal microscope, did not colocalize with chlorophyll autofluorescence, indicating that the N-terminal cTP is necessary for the proper localization of MFL proteins in plant cells.

### 2.4. Functional Characterization of MFL Proteins in Yeasts

**Figure 4:**
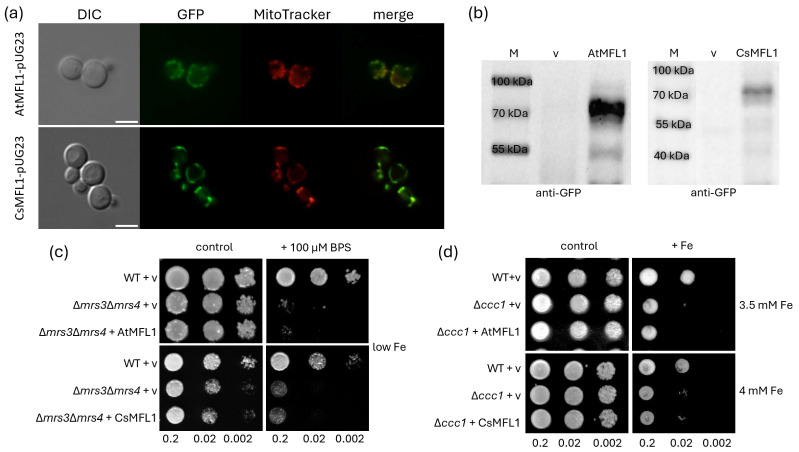
Characterization of AtMFL1 and CsMFL1 in yeast. Subcellular localization of AtMFL1 (AtMFL1-pUG23) and CsMFL1 (CsMFL1-pUG23) in yeast *Saccharomyces cerevisiae,* determined by fluorescence microscopy (**a**) and Western blot analysis (**b**). Green—GFP fluorescence, red—mitochondrial dye MitoTracker CMxRos, merge—combination of green and red signals. The scale bar corresponds to 5 μm. M—protein marker, v—pUG23 vector. Complementation assay of AtMFL1 and CsMFL1 on iron-sufficient (control) and iron-deficient (+100 μM BPS, low Fe) media (**c**). Complementation assay of AtMFL1 and CsMFL1 on iron-sufficient (control) and iron-excess (+Fe, 3.5 mM/4 mM Fe) media (**d**). Serial dilutions were spotted (OD600 of 0.2; 0.02; 0.002). v—pUG23 vector. The presented images are representative of at least 3 biological replicates.

Since MFL1 proteins are present in chloroplasts in planta, in a first step, their localization in yeast was analyzed to assess whether functional characterization can be performed in this heterologous system. Yeast cells were transformed with AtMFL1-pUG23 or CsMFL1-pUG23 vector and stained with the mitochondria-specific dye Mitotracker Red. As shown in Figure 4a, colocalization of both AtMFL1 and CsMFL1 (tagged with GFP) with Mitotracker Red indicates their targeting to yeast mitochondria. This was further confirmed by Western blot analysis with the yeast mitochondrial fractions and antibodies directed against GFP. The performed immunoblots showed proteins with a molecular weight of approximately 70 kDa, which was expected for the At/CsMFL1-GFP fusion protein (Figure 4b). Thus, it was found that MFL1s are not targeted to the cytoplasm, inclusion bodies, or for vacuolar degradation, and they can be further analyzed in yeast.

To evaluate the possible function of MFL1 proteins as iron importers, the aforementioned vectors were introduced into the Δ*mrs*Δ*3mrs4* double mutant, which is sensitive to low iron conditions due to the lack of high-affinity mitochondrial iron importers [29] (Figure 4c), as well as into the Δ*ccc1* mutant, which lacks vacuolar Fe/Mn iron importers [30], and for this reason, they are sensitive to high iron conditions (Figure 4d). Neither AtMFL1 nor CsMFL1 complemented the growth of the Δ*mrs3*Δ*mrs4* strain under iron-deficiency conditions (Figure 4c), nor the Δ*ccc1* strain under iron-excess conditions (Figure 4d). This excluded any further investigation into the role of plant MFL1s as iron importers in yeast.

### 2.5. Effects of AtMFL1 Overexpression on Arabidopsis Plants

**Figure 5:**
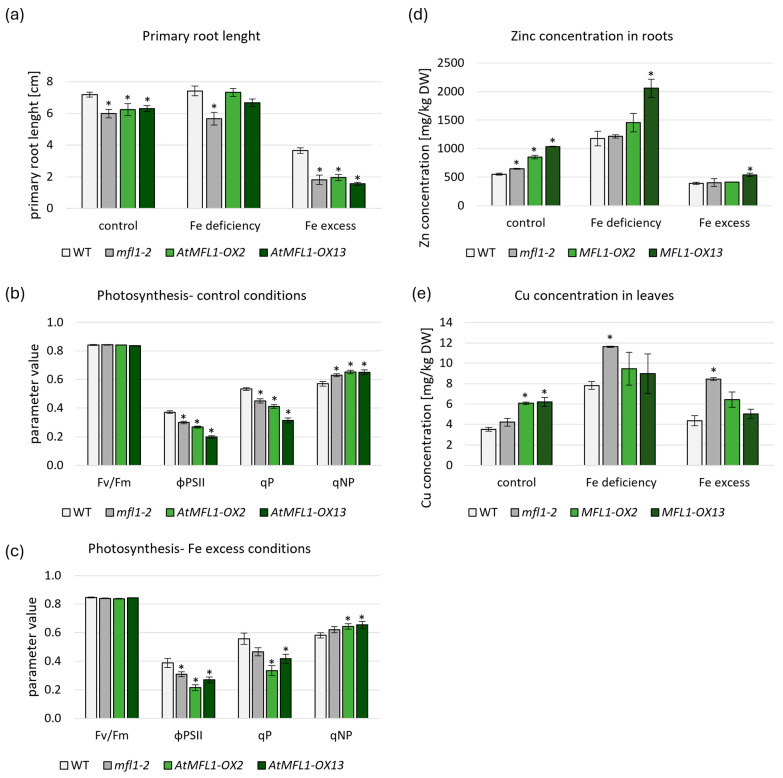
Characterization of *atmfl1-2* and *AtMFL1-OX* mutants. Primary root length of the 2-week-old wild-type, *atmfl1-2* mutant, and two *AtMFL1* overexpressing lines (**a**) grown under control conditions (control medium), Fe deficiency (medium without FeSO_4_–EDTA), and Fe excess (medium supplemented with FeSO_4_–EDTA to a final concentration of 0.2 mM). Photosynthesis parameters measured in the 8-week-old wild-type, *atmfl1-2* mutant, and in the two *AtMFL1* overexpressing lines grown for 2 weeks under control conditions (**b**), and iron-excess conditions (**c**)—medium supplemented with 0.2 mM FeSO_4_–EDTA. Fv/Fm—maximum quantum yield of PSII, ϕPSII—quantum yield of PSII, qP—photochemical fluorescence quenching, qNP—nonphotochemical quenching. Zn accumulation in roots (**d**) and Cu accumulation in leaves (**e**) measured in the 8-week-old wild-type, *atmfl1-2* mutant, and two *AtMFL1* overexpressing lines grown under control conditions (control medium), Fe deficiency (medium without FeSO_4_–EDTA with an added 0.05 mM BPS for two weeks), and Fe excess (medium supplemented with FeSO_4_–EDTA to a final concentration of 0.2 mM for two weeks). DW—dry weight. All presented values are the means of three separate biological replicates. Significance was assayed by Student’s *t*-test (* < 0.05). Asterisks represent statistically significant differences between WT and knockout or overexpression mutants.

To assess the possible function of MFL proteins in Arabidopsis, one knockout (KO) line (*atmfl1-2*) and two selected independent overexpression lines (OXs), with varied levels of *AtMFL1* overexpression (Figure S1), were used. AtMFL1 protein expression in the overexpressing lines was confirmed by immunoblotting with antibodies against a GFP tag (Figure S2). All experiments described in this paragraph were performed on plants growing under control, iron-deficiency, and iron-excess conditions. Primary root-length measurements of 2-week-old Arabidopsis seedlings showed that the root growth was negatively affected in the *atmfl1-2* mutant under all tested conditions compared to WT plants (Figure 5a). Similarly, root length was diminished in both overexpressing lines under control and Fe-excess conditions (Figure 5a). A negative effect of both gene knockout and overexpression on photosynthesis efficiency was also noted. Under control (Figure 5b) and Fe-excess conditions (Figure 5c), OX lines displayed lower PSII operating efficiency (ϕPS2II) and photochemical quenching (qP) levels, with simultaneous higher non-photochemical quenching (qNP). The same relationship was also observed for the KO line under control conditions (Figure 5b). Interestingly, no changes in photosynthesis parameters were found under Fe-deficiency conditions (Figure S3).

The accumulation of micronutrients in individual plant organs is considered to be an important factor in the characterization of transgenic lines. Measurements conducted in the roots and leaves of 8-week-old plants indicated only a small increase in Fe concentration in leaves of the *atmfl1-2* mutant compared to WT under control conditions, but no clear trend was found for overexpression lines (Table S1). Interesting observations were made during the measurements of Zn concentration under control conditions (Figure 5d and Table S2), which showed a significant increase in the accumulation of this micronutrient in the roots of *AtMFL1*-overexpressing lines. Similar results were obtained regarding Cu concentration in leaves under control conditions (Figure 5e and Table S2), demonstrating an increased accumulation in overexpressing lines compared to WT. An increase in Cu concentration was also noted in the leaves of the KO mutant under Fe-deficiency and Fe-excess conditions (Figure 5e). Mn concentration remained relatively stable in all tested plants, with the exception of the *atmfl1-2* mutant, which showed increased Mn accumulation in roots under control conditions (Table S1).

**Figure 6:**
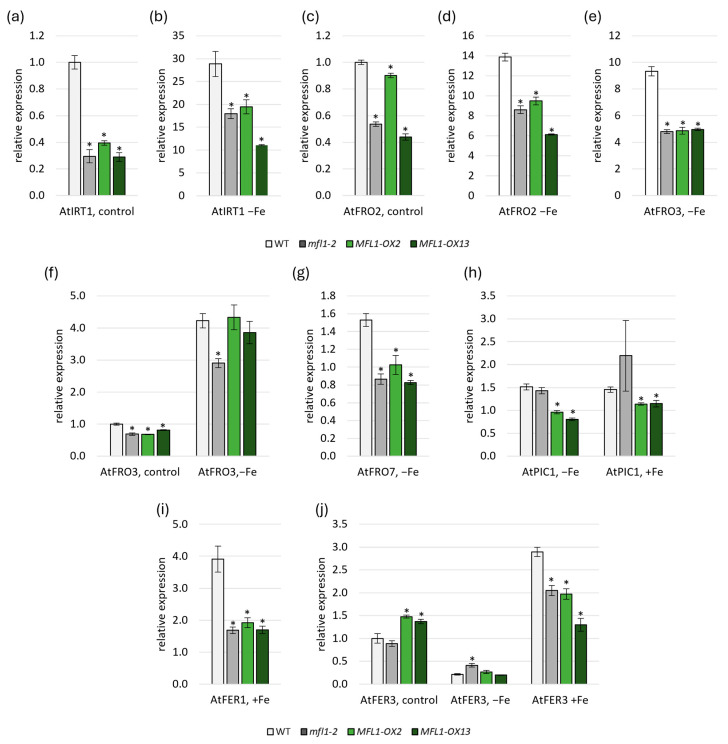
Expression of selected genes involved in iron homeostasis in *atmfl1-2* and *AtMFL1-OX* mutants. Gene expression analysis of *AtIRT1, AtFRO2,* and *AtFRO3* in roots (**a**–**e**) and *AtFRO3*, *AtFRO7*, *AtPIC1*, *AtFER1*, and *AtFER3* in leaves (**f**–**j**) of 8-week-old wild-type, *atmfl1-2* mutant, and two *AtMFL1* overexpressing lines under control conditions (control), Fe deficiency (−Fe, −FeSO_4_–EDTA, +0.05 mM BPS for two weeks), or Fe excess (+Fe; +0.2 mM FeSO_4_–EDTA for two weeks). The obtained values were calculated relative to the reference gene *AtCACS*, according to the ΔΔCT method. The presented results are the means of three biological replicates. Error bars represent standard error (±SE). Asterisks represent statistically significant differences between WT and knockout or overexpression mutants (*p* < 0.05; Student’s *t*-test).

Another noteworthy aspect to be studied was the analysis of the gene expression of proteins involved in iron homeostasis in plants, which was carried out in the *AtMFL1* knockout and overexpression lines (Figure 6, Tables S3 and S4). In roots, the expression of *AtIRT1* (Figure 6a,b) and *AtFRO2* (Figure 6c,d) involved in Fe uptake was lower in *atmfl1-2* mutant and *AtMFL1-OX* lines than in the wild type under both control and iron-deficiency conditions. A similar expression pattern was found for the mitochondrial metalloreductase *AtFRO3* under iron deficiency (Figure 6e). In leaves, downregulation of *AtFRO3* expression was also observed in the KO mutant under both control and iron-deficiency conditions, as well as in *AtMFL1-OX* lines under control conditions (Figure 6f). When analyzing the level of genes encoding chloroplast proteins involved in iron acquisition, it was found that the expression of *AtFRO7* responsible for iron reduction in chloroplasts was downregulated under iron-deficiency conditions in all analyzed lines compared to the wild type (Figure 6g). Interestingly, the expression of *AtPIC1* encoding the primary chloroplast iron importer was also reduced in *AtMFL1-OX* lines under both iron-deficiency and iron-excess conditions (Figure 6h). Moreover, the expression of genes encoding iron storage proteins AtFER1 (Figure 6i) and AtFER3 (Figure 6j) was lowered in all analyzed lines under iron-excess conditions, and additionally, the level of *AtFER3* was increased in the overexpression lines under control conditions and in the KO mutant under iron deficiency.

## 3. Discussion

Mitoferrins are a group of transporters belonging to the Mitochondrial Carrier Family of proteins [17]. They were first characterized in the yeast *Saccharomyces cerevisiae* as ScMRS3 and ScMRS4 [31] and then as mitoferrins in model animal species such as *Danio rerio* [32] or *Drosophila melanogaster* [33]. All aforementioned proteins localize to mitochondria and are involved in mitochondrial iron import. The first plant mitoferrins were identified and characterized in two model plants: OsMIT1 in rice [20] and AtMFL1 [14], as well AtMIT1 and AtMIT2 [21] later in Arabidopsis. Rice protein, like its animal homologs, functions as a mitochondrial iron importer, the knockout of which results in a lethal phenotype [20]. Similarly, AtMIT1 and AtMIT2 are high-affinity mitochondrial iron importers, and the simultaneous knockout of both proteins is also lethal [21]. Surprisingly, in silico analysis of the Arabidopsis MFL1, identified by searching for sequences homologous to mitoferrin 2 of *Danio rerio* [14], as well as proteomic data [34,35], suggested that it localizes to chloroplasts. Moreover, based on the phylogenetic data, it was proposed that it clusters evolutionarily with the aforementioned mitoferrin 2 and not with mitoferrin 1 [36]. To identify homologs of AtMFL1 in cucumber, the genomic DNA contigs of *C. sativus* cultivar Borszczagowski were searched. One potential sequence was found in the ACYN01002741.1 contig and named *CsMFL1*. AtMFL1 and CsMFL1 share 67% homology with each other and around 25–30% homology with plant MIT proteins. According to the theory presented by Haferkamp and Schmitz-Esser [17], the MFL gene could have arisen as a result of duplication of the MIT sequence in plants. However, due to the potentially different subcellular localization than the MIT proteins and a possible change in function, a number of modifications have occurred within the gene sequence, resulting in the lower percentage of homology to the MITs.

Since the chloroplast localization of mitoferrin-like proteins is mainly based on in silico analysis and proteomic data, in this study, we decided to verify their localization in *A. thaliana* protoplasts. The obtained results confirmed the presence of both AtMFL1 and CsMFL1 proteins in chloroplasts. Proteomic analyses of these organelles in other plant representatives, such as pea [37], maize [38], or cauliflower [39], also indicated that homologs of the analyzed MFLs may localize in plastids, suggesting that the subcellular localization of this group of proteins is specific for plants. According to the in silico analysis of the protein sequences, MITs do not possess a cleavable targeting sequence at the N-terminus, but as proteins of the inner mitochondrial membrane, they may contain a type of signal peptide within their protein structure [40]. In contrast to the MIT proteins, a putative 92 amino acid N-terminal targeting sequence was identified for AtMFL1 and, by homology analysis, a corresponding 79 amino acid N-terminal targeting peptide was found in CsMFL1. Truncated versions of both mitoferrin-like proteins, lacking the predicted chloroplast targeting sequence, have been mislocalized, confirming the necessity of this type of signal peptide for the proper localization of MFL proteins in plant cells. Plants, as photosynthetic organisms containing both chloroplasts and mitochondria, must precisely target proteins to both of these organelles. Some proteins of the mitochondrial inner membrane (such as MITs) possess targeting sequences located within their protein structure, while most of the mitochondrial proteins harbor the N-terminal targeting sequence [41]. Therefore, such N-terminal signal peptides, directing proteins to chloroplasts and mitochondria, have two functional domains: one (N-terminal) responsible for the specificity of the subcellular localization of a given protein, and the other (C-terminal) containing motifs supporting protein translocation across either chloroplast or mitochondrial membranes [42].

All mitoferrins/MITs characterized so far from yeast, animals, and plants are mitochondrial iron importers. Mitoferrin-like proteins have also been suggested to transport Fe ions [14]. The literature lacks broader studies devoted to the analysis of *MFL* gene expression in individual plant organs, especially under various iron accessibility. The available data, provided by the Arabidopsis eFP Browser 2.0 database [43], indicates a higher expression of *AtMFL1* in leaves than in roots. The only experimental data on the expression of *AtMFL1* comes from the analysis of 6-day-old whole Arabidopsis seedlings, which shows that it is Fe-dependent [14]. The research conducted as a part of this work confirmed the previous data and demonstrated gene expression in shoots more than six times higher than that in roots of 8-week-old Arabidopsis plants. On the other hand, the *CsMFL1* expression revealed a different profile in cucumber. In contrast to Arabidopsis, no significant differences were noted between the vegetative organs analyzed, especially between roots and leaves. Analyzing the effect of iron deficiency and excess on *MFL1* expression, further discrepancy between *AtMFL1* and *CsMFL1* has emerged. Tarantino et al. [14] showed that *AtMFL1* expression was higher under Fe excess in comparison to the control conditions in 6-day-old seedlings. In this work, we confirmed a similar Fe-dependent relationship in 8-week-old Arabidopsis leaves. Interestingly, high iron conditions had a much more pronounced effect after 24 h of treatment compared to 2 weeks, which may suggest the strongest plant response in the early phases of exposure to stress. Iron deficiency had the opposite effect in two respects. *AtMFL1* expression was downregulated in leaves of 8-week-old plants, and the observed inhibition was more pronounced under a long-term treatment. Analogous analysis of cucumber *CsMFL1* expression showed changes only in roots but not in leaves. *CsMFL1* transcript levels were upregulated under both short- and long-term iron-deficiency conditions, with no effect found under iron-excess conditions. This pattern of gene expression suggests that the function of the MFL1 protein in both Arabidopsis and cucumber may be affected by the availability of iron in the environment. This may also imply that model and crop plants activate different iron-related adaptation mechanisms. In Arabidopsis, due to the elevated gene expression, it can be hypothesized that AtMFL1 is responsible for iron transport and storage in leaf chloroplasts, predominantly under Fe-excess conditions. Conversely, under Fe-deficiency conditions, its function does not seem to be essential, which is associated with reduced transcript accumulation. In cucumber, the MFL1 function appears to be more versatile. The lack of significant alterations in its gene expression suggests that this protein may facilitate iron transport to plastids under varying iron availability conditions in both roots and leaves. It is particularly intriguing that it may also be responsible for the regulation of iron homeostasis or redistribution in roots under Fe deficiency.

However, it should also be noted that most of the indicated changes in *AtMFL1* and *CsMFL1* expression were within the range of twofold change, which is on the border of being biologically significant [44]. Nonetheless, the obtained results showed clear differences in the *MFL* expression profiles between the model plant and the crop plant. Unfortunately, the lack of comparative data for other plant species does not allow for the establishment and formulation of universal final conclusions regarding *MFL* expression under a different Fe regime. This requires further studies in the future.

As mentioned earlier, the homology of MFLs to mitoferrins and the upregulation of their gene expression in response to iron excess, as observed in Arabidopsis, may suggest that these proteins are iron transporters. The heterologous yeast system is a popular method to analyze the substrate specificity of plant proteins, including those localizing to chloroplasts. In yeast cells, both MFL1 proteins localized to mitochondria. When chloroplast proteins are synthesized in cells or organisms lacking plastids, they are often directed to mitochondria. For this reason, the obtained results may be due to the inability of yeast to distinguish chloroplasts from mitochondrial sequences [45,46]. Nevertheless, we were able to analyze the possible function of MFL1 proteins as iron transporters in two yeast strains: one lacking the high-affinity mitochondrial iron importers ScMRS3 and ScMRS4 [31], and the other lacking the vacuolar Fe/Mn transporter ScCCC1 [30]. The ∆*mrs3*∆*mrs4* mutation leads to impaired yeast growth under iron-deficiency conditions, which could be rescued by other high-affinity iron importers. This technique was routinely used to study mitoferrin/MIT function [20,21,23]. As our results indicate, neither AtMFL1 nor CsMFL1 complemented this phenotype. On this basis, it can be assumed that MFLs do not exhibit a high affinity for iron, and in chloroplasts, they do not perform a function analogous to that of mitochondrial MIT proteins, which was also postulated by Tarantino et al. [14]. In *A. thaliana*, AtPIC1 permease is the primary importer of iron into chloroplasts [11]. Tarantino et al. [14] postulated a supporting role of the MFL1 protein in this process, because an Arabidopsis mutant lacking this protein did not show any clear phenotypic changes under control conditions. The only noticeable phenotypic symptoms appeared in 21-day-old plants, which were characterized by limited rosette growth under Fe-excess conditions. Following this line of reasoning, we then used the ∆*ccc1* yeast strain, which exhibits high cytosolic Fe accumulation, making it sensitive to high iron concentrations in the medium. High cytosolic Fe should result in its transport to other cellular compartments to at least partially prevent the toxic metal effects in the cytoplasm. This effect was observed in the case of overexpression of the low-affinity mitochondrial transporter ScRIM2 in the Δ*ccc1* strain [47,48]. In our studies, AtMFL1 and CsMFL1 did not complement the ∆*ccc1* phenotype. Additionally, AtMFL1 was also unable to complement the growth of the yeast ∆*ftr3* mutant, which lacks the plasma membrane Fe^3+^ importer [49]. As the authors pointed out, this may indicate that Fe^3+^ is not a preferred substrate for mitoferrin-like transporters [49]. On the other hand, this may suggest that MFL1 proteins do not function properly in yeast. What is more, it can also suggest that these proteins are neither high-affinity nor low-affinity iron importers. An alternative hypothesis regarding the function of MFLs, proposed by Tarantino et al. [14], assumes that they are Fe exporters and that their function is regulated depending on the chloroplast demand for this microelement [14].

To provide a more comprehensive characterization of the AtMFL1, we decided to investigate the effect of its knockout and overexpression in Arabidopsis plants. Iron deficiency is a growth-limiting factor that affects around 30% of arable lands due to their neutral or alkaline pH [50]. Studying the effects of knockout or overexpression of proteins involved in iron homeostasis in dicotyledonous plants is an important step towards deciphering the precise mechanisms of iron-level regulation both in Fe-rich organelles such as chloroplasts and in the whole organism. Characterization of the *AtMFL1* knockout mutant by Tarantino et al. [14] revealed that 21-day-old seedlings exhibited reduced growth and slightly diminished maximal photochemical efficiency of PSII (Fv/Fm). In this work, the growth of primary roots and leaf rosettes was reduced compared to wild-type plants in 2-week-old seedlings of the *atfml1-2* mutant grown under control, Fe-deficiency, or Fe-excess conditions. Photosynthesis parameters of 8-week-old plants, such as ϕPSII, qP, and qNP, were also negatively affected under control and Fe-excess conditions. Our observations therefore confirmed the results obtained by Tarantino et al. [14] that AtMFL1 is probably involved in maintaining iron homeostasis but not as an essential protein, since the mutant plants remained viable throughout their vegetative growth period and none of the measured parameters changed drastically.

Overexpression of primary chloroplast iron importer permease *PIC1* leads to leaf chlorosis and significantly affects plant growth due to iron overload in chloroplasts [51]. In plants overexpressing *AtMFL1*, no leaf chlorosis was observed under any of the tested conditions, with the greatest reduction in root length noted under Fe excess and a smaller one under control conditions. In contrast, no effects were observed in plants under iron deficiency. Similarly, when analyzing photosynthesis parameters, negative effects of overexpression were observed only under control and Fe-excess conditions, but not under iron deficiency. Improper iron homeostasis in cells can lead to an imbalance and the generation of ROS via the Fenton reaction [52], which in turn can lower photosynthesis efficiency [53]. Additionally, the response of plants to perceived iron overload may be a reduction in root growth [54], which was observed in *MFL1-OX* lines. It is worth noting that homozygous *PIC1-OX* lines were not viable [51], while *AtMFL1* overexpression had overall adverse but much smaller effects on growth and photosynthesis parameters of Arabidopsis plants under control and iron-excess conditions. Therefore, it appears that AtMFL1 function, although related to Fe homeostasis, is either different to that of AtPIC1 or regulated in an alternative manner.

Since the MFL1 participates in maintaining iron homeostasis, changes in its level can affect the functioning of other proteins involved in this process. For this reason, we analyzed the gene expression level of iron acquisition proteins AtIRT1 and AtFRO2 [55,56,57], intracellular ferric chelate reductases (FRO3/6/7/8) [58,59], iron transporters AtFPN3 [60] and AtPIC1 [11,51], and iron storage proteins AtFER1/3/4 [61] in both the roots and the leaves of 8-week-old *AtMFL1* knockout and overexpression lines. Deregulated expression of genes related to iron homeostasis has been demonstrated to be a hallmark of both knockout and overexpression mutants of chloroplast iron transporters [51,60]. A previous study by Tarantino et al. [14] showed that the expression of neither mitochondrial reductases *AtFRO3* and *AtFRO8* nor chloroplast reductase *AtFRO7* was altered in 6-day-old seedlings of the *atmfl1-2* mutant grown under iron-sufficient, -deficient, and -excess conditions. In turn, the expression of *AtFER1* was reduced when both sufficient and excess iron was supplied to the medium. The results obtained in this study indicated that generally, in both roots and leaves of the 8-week-old knockout mutant, the expression pattern of *AtFRO3*, *AtFRO7*, and *AtFRO8* was similar in older plants, with some changes noted mainly under iron-deficiency conditions. Moreover, the expression of *AtFER1* was diminished under control and iron-excess conditions in the knockout mutant, but contrary to Tarantino et al. [14], under iron deficiency, the abundance of *AtFER1* transcripts was markedly higher. These results seem to indicate that the *AtFER1* level may be differently regulated in the *atmfl1-2* young seedlings and mature plants. The phenomenon of disparate gene regulation in younger and older plants has been previously demonstrated for genes associated with iron homeostasis [62].

Similarly to *AtFER1*, the expression of *AtFER3* and *AtFER4* was also higher in leaves of 8-week-old iron-deficient Arabidopsis mutants compared to wild-type plants and lower under control conditions (*AtFER3* in roots) or iron-excess conditions (*AtFER4* in leaves). Since ferritin expression is induced by Fe [63], it is possible that the chloroplast iron status is higher in the *atmfl1-2* mutant than in wild-type plants under iron deficiency. Alternatively, increased expression of ferritins may result from elevated oxidative stress in the mutant’s chloroplasts due to disturbances in iron homeostasis [61]. The *AtMFL1-OX* lines generated in this study showed a distinct ferritin expression pattern, with downregulation of *FER1* and *FER3* under iron-excess conditions. Notably, the downregulation of *FER1* and *FER3* in *AtMFL1-OX* lines, as well as of *FER1* in the *atmfl1-2* knockout mutant, demonstrated similarities to the phenotypes characteristic of *AtPIC1-OX* lines observed under control conditions [51] and the *fpn3* knockdown mutant [60]. This may suggest a potential similarity of both *AtMFL1* overexpression and disruption to the aforementioned mutants, where the mutation leads to oxidative stress in plants due to iron homeostasis disturbances in chloroplasts and whole cells.

Another noteworthy result seems to be the decreased expression of iron acquisition genes *AtIRT1* and *AtFRO2* in roots of the *atmfl1-2* mutant under both control (*AtIRT1*, *AtFRO2*) and iron-deficient conditions (*AtIRT1*, *AtFRO2*). In *AtMFL1-OX* lines, reduced expression of these genes was also observed under control (*AtIRT1*) and iron-deficient conditions (*AtIRT1*, *AtFRO2*). Although this result may suggest a higher perceived *in planta* iron concentration [55,56], it is also important to note that both proteins are regulated at the post-transcriptional and post-translational level by the Fe status in roots and shoots, and by the ratio of other micronutrients to iron [55,56,64,65,66]. Thus, on one hand, since *AtMFL1* is expressed in roots, the observed changes may indicate local disturbances in *AtMFL1* activity and, consequently, in iron homeostasis in this specific tissue. Alternatively, they may be the result of a shoot-borne signal, as iron homeostasis regulation is a result of both local and systemic signaling [67]. This is particularly evident in the *AtMFL1-OX* lines, which exhibited a distinct gene expression pattern in leaves. This pattern was characterized by reduced transcript levels of genes encoding proteins responsible for iron reduction on the plasma membrane (*AtFRO6*) and iron acquisition into chloroplasts (*AtFRO7*, *AtPIC1*) under Fe-deficiency conditions. All three genes are indispensable for transporting iron into cells and chloroplasts. Therefore, alterations in their expression levels in lines overexpressing *AtMFL1* suggest a potential role for AtMFL1 as an iron transporter or a protein regulating iron homeostasis. Although the changes in the expression of iron homeostasis genes observed in this study were small, they imply that knocking out or overexpressing *AtMFL1* leads to disruptions in cellular iron homeostasis in root and leaf cells, possibly due to AtMFL1 involvement in the process of chloroplast iron transport.

Analysis that focuses solely on changes in gene expression does not provide a comprehensive understanding of the regulatory mechanisms governing metal homeostasis throughout the plant. For this purpose, measurements of the accumulation of essential elements are carried out. In this particular case, Fe, Zn, and Mn levels remained relatively constant in the roots and shoots of the 8-week-old *atmfl1-2* mutant compared to wild-type plants under all tested conditions. Similar results were obtained for Fe and Mn accumulation in *AtMFL1-OX* lines. Interestingly, under iron deficiency and excess, Cu accumulation in leaves was higher in the knockout mutant. Elevated levels of zinc in the roots and copper in the leaves were also observed in the *AtMFL1-OX* lines. The higher accumulation of Cu and Zn in the mutants may therefore indicate a disruption in iron homeostasis. One of the most well-documented hallmarks of iron–copper crosstalk is overaccumulation of one element in the absence of the other. Additionally, the presence of both microelements within the cells is subjected to tight regulation [68,69,70,71]. These results further substantiate the concept that the homeostasis of iron and other essential metals in plants is governed by multiple regulatory mechanisms pertaining to their abundance in organelles, cells, and, finally, whole plants. Crosstalk between iron and other micro- and macronutrients has emerged as a pivotal area of research interest, prompting intensive studies in this field [70,72].

In summary, the findings presented in this study indicate that AtMFL1 and CsMFL1 may function as chloroplast iron transporters, whose gene expression is regulated by iron availability. The differences in amino acid residues, designated as substrate contact points or iron-binding sites [18,28], between mitoferrins and mitoferrin-like proteins suggest that distinct mechanisms of substrate transfer exist for plastid and mitochondrial transporters. On the one hand, the results obtained, consistent with those reported by Tarantino et al. [14], support the hypothesis that *AtMFL1* is not an essential gene for Arabidopsis. On the other hand, however, they point to a potential involvement of mitoferrin-like proteins in the regulation of cellular iron homeostasis and the modulation of iron fluxes to chloroplasts via Fe import and/or export. Considering that micronutrient deficiency, especially Fe shortage, constitutes a major challenge to agriculture [50], studies including mutants of genes encoding proteins involved in metal homeostasis represent an important research avenue for plant biofortification. Knockout or overexpression of only *AtMFL1* did not significantly affect the expression of many genes involved in iron homeostasis, or the accumulation of micronutrients such as Fe, Zn, Cu, and Mn. Nonetheless, the observed alterations in the expression of genes encoding proteins involved in iron acquisition, intracellular trafficking, and, most notably, Fe storage in chloroplasts, as well as changes in copper and zinc levels, suggest that modified *MFL1* expression, in combination with changes in gene expression of other proteins, could potentially result in improved acquisition and accumulation of the key micronutrients. This research pathway is of particular interest for studies on dicotyledonous plants. Similar strategies, i.e., combining the overexpression of several different proteins, have been successfully applied in biofortification studies of various grasses [73].

## 4. Materials and Methods

### 4.1. Plant Growth Conditions

To analyze cucumber gene expression, *Cucumis sativus* (var. Krak) seedlings were grown on control hydroponic media as described by Migocka et al. [74], with some modifications: for iron deficiency, the control medium was prepared without FeSO_4_–EDTA and with the addition of 30 µM BPS (bathophenanthroline disulfonate, Sigma, St. Louis, MO, USA); for iron excess, the control medium was supplemented with FeSO_4_–EDTA to a final concentration of 1 mM. Plants were grown at 24 °C under a 16 h/8 h day/night photoperiod (180 µmol m^−2^ s^−1^). For Arabidopsis gene expression and heavy metal content assays, seeds of wild-type (WT) Columbia ecotype (Col-0) and transgenic lines (*atmfl1-2,* SALK 007671, and overexpressing lines obtained in this work) were surface-sterilized, placed on the half-strength MS (Murashie and Skoog) medium (Sigma) with 0.05% (*w*/*v*) MES and 0.65% (*w*/*v*) agar in the 0.5 mL plastic tubes with a cut bottom, and stratified in the dark at 4 °C. After 2 days, tubes were placed in a hydroponic system and plants were grown for a total of 8 weeks at 22 °C under an 8 h/16 h day/night photoperiod (120 µmol m^−2^ s^−1^) in a control medium as described by Morel et al. [75], with some modifications: for iron deficiency, the control medium was prepared without FeSO_4_–EDTA and with the addition of 50 µM BPS; for iron excess, the control medium was supplemented with FeSO_4_–EDTA to a final concentration of 200 µM. In both cases, 24 h and 2-week treatments of Fe deficiency and excess were applied.

### 4.2. Arabidopsis Primary Root Measurements

Root measurements were carried out on 2-week-old Arabidopsis plants. Surface-sterilized seeds were placed on plates containing a medium prepared as described by Gollhofer et al. [76], with some modifications: for iron deficiency, the medium was prepared without FeSO_4_–EDTA, while for iron excess, the medium was prepared with FeSO_4_–EDTA added to a final concentration of 200 µM. Plates were first placed in the dark at 4 °C for 2 days and then at 23 °C under a 16 h/8 h day/night photoperiod (120 µmol m^−2^ s^−1^). Primary root length was determined using Fiji software (v2.15.1) [77].

### 4.3. Complementation Assays and Fluorescent Imaging in Yeast

The yeast strains used in this study were ∆*mrs3*∆*mrs4* [78] with isogenic wild-type strain DY150 [78] and ∆*ccc1* (Euroscarf, Oberursel, Germany) with isogenic wild-type strain BY4742 [79]. Wild-type strains were transformed with an empty pUG23 vector as a positive control [80], while mutant strains were transformed with either the empty pUG23 vector (negative control) or the AtMFL1/CsMFL1-pUG23 vector. The yeasts were selected on plates with an SC medium containing a 0.67% (*w*/*v*) BD Difco^TM^ yeast nitrogen base without amino acids (BD—Becton, Dickinson and Company, Franklin Lakes, NJ, USA, 2% (*w*/*v*) glucose, and 2% (*w*/*v*) agar, and supplemented with amino acids without histidine (SC-His). Complementation assays were carried out using yeast cells grown overnight, with serial dilutions spotted on agar plates (SC-His) with 0.1 mM BPS added for iron deficiency or 3.5 mM (AtMFL1) or 4 mM (CsMFL1) FeSO_4_–EDTA added for iron excess. Plates were incubated at 30 °C for 3–5 days. For protein localization, an overnight yeast culture was incubated for 60 min with 100 nM of the mitochondria-specific dye MitoTracker Red CMXRos (Invitrogen, Waltham, MA, USA), and the images were acquired using an Axio Imager M2 Fluorescence Microscope (Carl Zeiss, Oberkochen, Germany).

### 4.4. Gene Expression Analysis

Roots, hypocotyls, cotyledons, and first leaves of 2-week-old cucumber seedlings and whole 2-week-old Arabidopsis seedlings, as well as roots and rosette leaves of 8-week-old Arabidopsis plants, were collected for gene expression analysis. RNA was isolated according to the manufacturer’s instructions with Extrazol (Blirt, Gdansk, Poland), and its concentration was measured using NanoDrop ND-1000 (Thermo Fisher Scientific, Waltham, MA, USA). Total RNA was treated with DNase I (Thermo Fisher Scientific) and reverse-transcribed using the High-Capacity cDNA Reverse Transcription Kit (Applied Biosystems, Waltham, MA, USA), according to the manufacturer’s instructions. Gene expression was assayed by real-time PCR in a Lightcycler 480 (Roche, Basel, Switzerland) using the RealTime 2× PCR SYBR Mix (A&A Biotechnology, Gdansk, Poland). The primers used are listed in Table S5, and their specificity was checked by a melting curve analysis. In both Arabidopsis and cucumber, the presented results were normalized to the reference gene encoding the clathrin adaptor complex subunit (AtCACS/CsCACS) using the ΔΔCT method [81,82,83]. The presented values are the means of 3 biological replicates.

### 4.5. Cloning into Expression Vectors

*AtMFL1* and *CsMFL1* sequences were amplified by PCR using the cDNA template obtained from 8-week-old Arabidopsis or 2-week-old cucumber plants. The primers were designed based on sequences available in GeneBank (NCBI). The Arabidopsis gene sequence was available under accession number At5g42130. The *CsMFL1* sequence was identified in the ACYN01002741.1 contig of cucumber cultivar Borszczagowski by homology search (blastn, NCBI) using the *AtMFL* sequence as a template. The sequence of the cucumber homologue was submitted to GenBank under accession number OP454142.1. For the functional characterization in yeast, the sequences were cloned into SpeI-SalI sites of the pUG23 vector [80]. For protein localization in *A. thaliana* protoplasts, the sequences were cloned into SalI-SpeI sites of the pA7 vector [84]. To study the effect of *AtMFL1* overexpression in plants, the sequence of Arabidopsis cDNA was cloned using BP and LR clonases (Invitrogen) first into the pDONR^TM^221 vector (Invitrogen) and then into destination vector pMDC83 [85]. All aforementioned vectors ensure constitutive expression of proteins with a GFP tag at their C-terminus. The primers used for cloning are listed in Table S5. Vector sequences were confirmed by sequencing (Genomed, Warsaw, Poland).

### 4.6. Protoplast Transformation and Confocal Imaging

For subcellular localization of MFL1 proteins in plants, protoplasts were isolated from the *A. thaliana* cell suspension culture and used for transformation with AtMFL1-pA7 and CsMFL1-pA7 constructs as described previously [86]. Confocal imaging was performed using a Leica TCS-SP8 inverted confocal laser scanning microscope (Leica Microsystems, Wetzlar, Germany) 24–36 h after transformation.

### 4.7. Isolation of Subcellular Fractions from Yeast and Plants

Isolation of yeast mitochondria was carried out according to the procedure described by Gregg et al. [87], with one modification: prior to extraction, yeast cultures were grown for 48 h in SC-His medium. Chloroplast isolation from rosette leaves of 8-week-old Arabidopsis plants was carried out according to the procedure described by Besagni et al. [88], with some modifications: plants were not incubated in the dark prior to isolation; after homogenization, the extracts were first centrifuged for 10 min at 600× *g* to pellet nuclei, and then the obtained supernatants were centrifuged for 10 min at 1075× *g* to pellet chloroplasts; and the procedure was stopped after chloroplasts were obtained. The isolated fractions were used for Western blot analysis.

### 4.8. Protein Determination and Immunoblotting

Before Western blotting, the protein concentration in isolated fractions was measured by the Bradford assay [89]. A total of 20 µg of protein from each sample was used for the analysis. The presence of MFL1 proteins in yeast mitochondrial fractions as well as in plant chloroplast fractions was confirmed using antibodies against GFP (1:5000, Roche).

### 4.9. Measurements of Photosynthesis Parameters

Photosynthesis parameters were measured in 8-week-old Arabidopsis plants using an FMS2 fluorometer (Hansatech Instruments, Pentney, UK) according to the method described by Burzyński and Żurek [90]. Before each measurement, leaves were adapted in the dark for 20 min. The presented values are the mean (±standard error, SE) of 3 biological replicates.

### 4.10. Determination of Metal Content in Plants

The concentration of microelements (Fe, Cu, Zn, and Mn) in the roots and leaves of 8-week-old Arabidopsis plants was determined using a GBC Avanta PM Atomic Absorption Spectrometer (GBC Scientific, Keysborough, Australia). The collected plant organs were washed with deionized water to remove residual medium and dried at 80 °C for 2 days. The grounded tissues (100 mg of shoots; ca. 40 mg of roots) were digested in 10 mL pure 65% concentrated HNO_3_ (Merck KGaA, Darmstadt, Germany) with the addition of H_2_O_2_ (30% ultra-pure, Sigma). Measurements were carried out in samples diluted with deionized water to a volume of 10 mL (roots) or 25 mL (leaves). The concentrations of elements were analyzed against AA Standard Solutions (Sigma Chemical Co., St. Louis, MO, USA) and blanks containing deionized water and subjected to the same procedure as the samples. The accuracy of the determination was checked by analysis with Certified Reference Materials: IC-INCT-OBTL-5 (Oriental basma tobacco leaves, LGC, Middlesex, UK). The recovery rates were in the range of 94–106%. The content of metals in plants was calculated on a dry-weight basis.

### 4.11. Obtaining AtMFL1 Overexpressing Lines

Arabidopsis transformation of wild-type plants was carried out using the floral-dip method [91]. Plants resistant to the antibiotic were selected on 1/2 MS medium with 20 µM hygromycin as described previously [92]. The presence of the transgene was confirmed by PCR with DNA extracted from the T1 generation plants using the CTAB (cetyltrimethylammonium bromide) method. Segregation analysis of the T2 and T3 generations was used to obtain homozygous single insert lines. The T4 generation overexpression lines were designated *AtMFL1-OX* and the level of *AtMFL1* overexpression was determined by real-time PCR as previously described.

### 4.12. Bioinformatics and Statistical Analysis

Statistica^TM^ software version 14.0.0.15 (StatSoft, Krakow, Poland) was used for all statistical analyses. Normal distribution of data was determined by Shapiro–Wilk test, and homogeneity of variance was assessed by Levene’s test. Student’s t-test was used to determine the difference between two independent groups. Tukey’s test and analysis of variance (ANOVA) were used to assess the differences between more than two groups. Putative transmembrane domains in MFL proteins were predicted using CCTOP (v1.1.0) [93] and visualized using Protter (v1.0) [94]. N-terminal chloroplast transit peptide sequences were predicted using ChloroP (v1.1) [95].

## 5. Conclusions

**Figure 7:**
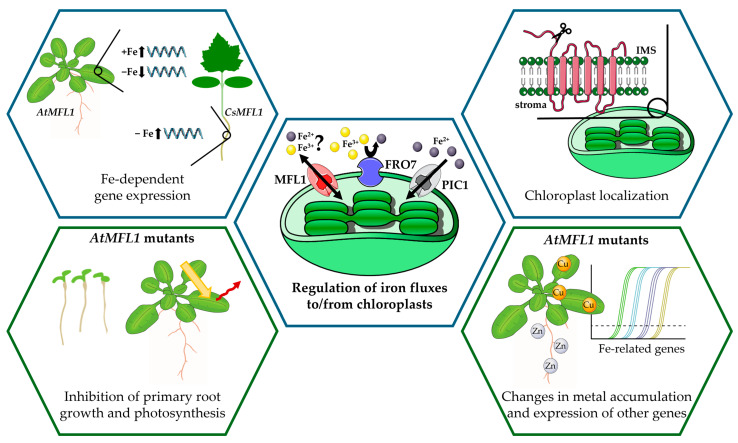
Functional characterization of MFL1 proteins from *Arabidopsis thaliana* and *Cucumis sativus*. The figure was prepared using adapted images from Servier Medical Art Database, Bioicons, and Openclipart. Servier Medical Art (https://smart.servier.com/, accessed on 16 July 2025) is licensed under CC BY 4.0 (https://creativecommons.org/licenses/by/4.0/). Images used from Bioicons (https://bioicons.com/, accessed on 16 July 2025) and Openclipart (https://openclipart.org/, accessed on 16 July 2025) are licensed under Creative Commons Zero 1.0 Public Domain License. −Fe—iron deficiency; +Fe—iron excess; IMS—intermembrane space.

Taken together, the results presented in this work indicate that AtMFL1 and CsMFL1 may function as chloroplast iron transporters, as was suggested by Tarantino et al. [14]. The observed relationship between their gene expression and iron availability is consistent with the proposed hypothesis that the primary iron transport pathway to chloroplasts is facilitated by PIC1, with MFL1 serving as an alternative route activated under specific conditions (Figure 7). It is worth emphasizing that the regulatory mechanisms may differ between model and crop plants. Similar differences between species, which are well documented in the literature, highlight the need for a dual focus of practice-oriented research, i.e., including both model and crop plants. Another important direction for future research seems to be the analysis of MFL1 protein expression under variable iron conditions, which may provide new insight into the role of this transporter in different plant species. Following this line of reasoning, amino acid residues designated as substrate contact points and iron-binding sites [18,28] in mitoferrins have not been found in mitoferrin-like proteins. This suggests either a different substrate or a transport mechanism for plastid and mitochondrial proteins belonging to the same group. Elucidating these mechanisms using, for example, site-directed mutagenesis may be crucial to understanding the complex network of chloroplast iron transport. Furthermore, studies using both knockout and overexpression mutants, as in this work, are helpful in determining the role of individual proteins in the cell and the whole plant. Although *AtMFL1* mutations did not significantly affect the physiology of the Arabidopsis plants, the changes noted suggest a role of AtMFL1 in overall iron management in plants, probably dependent on retrograding signaling from chloroplasts [6]. Therefore, while mutations in the *MFL1* gene do not appear to be particularly beneficial for plants, the question remains of whether combining them with mutations in genes of other proteins may yield more promising results.

## Data Availability

The data presented are available in this manuscript and the Supplementary Materials.

## Supplementary Materials

The following supporting information can be downloaded at: https://www.mdpi.com/article/10.3390/ijms26157103/s1. References [58,60,61,82,83,96,97,98] are cited in Supplementary Materials.
