# Supplementary material for: What Is Similar, What Is Different? Characterization of Mitoferrin-like Proteins from Arabidopsis thaliana and Cucumis sativus
Source: Int J Mol Sci. 2025 Jul 23;26(15):7103. doi: 10.3390/ijms26157103 (PMC12345680; doi:10.3390/ijms26157103)
Supplement: Supplementary file 1 [file ijms-26-07103-s001.zip › ijms-3767120-supplementary.pdf]

# What Is Similar, What Is Different? Characterization of Mitoferrin-like Proteins from *Arabidopsis thaliana* and *Cucumis sativus*

**Karolina Małas [1], Ludmiła Polechońska [2] and Katarzyna Kabała [1,*]**

[1] Department of Plant Molecular Physiology, Faculty of Biological Sciences, University of Wrocław, Kanonia 6/8, 50-328 Wroclaw, Poland; karolina.malas2@uwr.edu.pl

[2] Department of Ecology, Biogeochemistry and Environmental Protection, Faculty of Biological Sciences, Uni-versity of Wrocław, Kanonia 6/8, 50-328 Wroclaw, Poland; ludmila.polechonska@uwr.edu.pl

* Correspondence: katarzyna.kabala@uwr.edu.pl

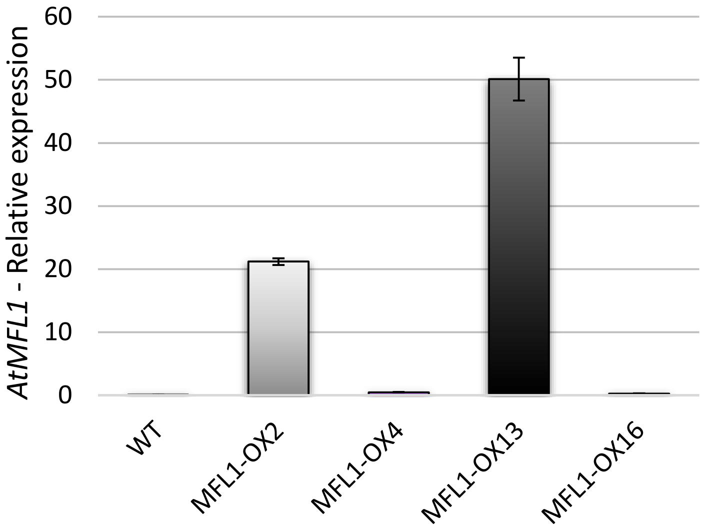


**Figure S1.** Expression of *AtMFL1* in overexpression lines.

Arabidopsis lines with *AtMFL1* overexpression were designated as *AtMFL1-OX1-20*. Further analyses were carried out using two of the lines selected: *AtMFL1-OX2* and *AtMFL1-OX13*. Gene expression was calculated relative to the reference gene *AtCACS,* according to the ΔΔCT method. Presented results are the means of three biological replicates. Error bars represent standard error (±SE).

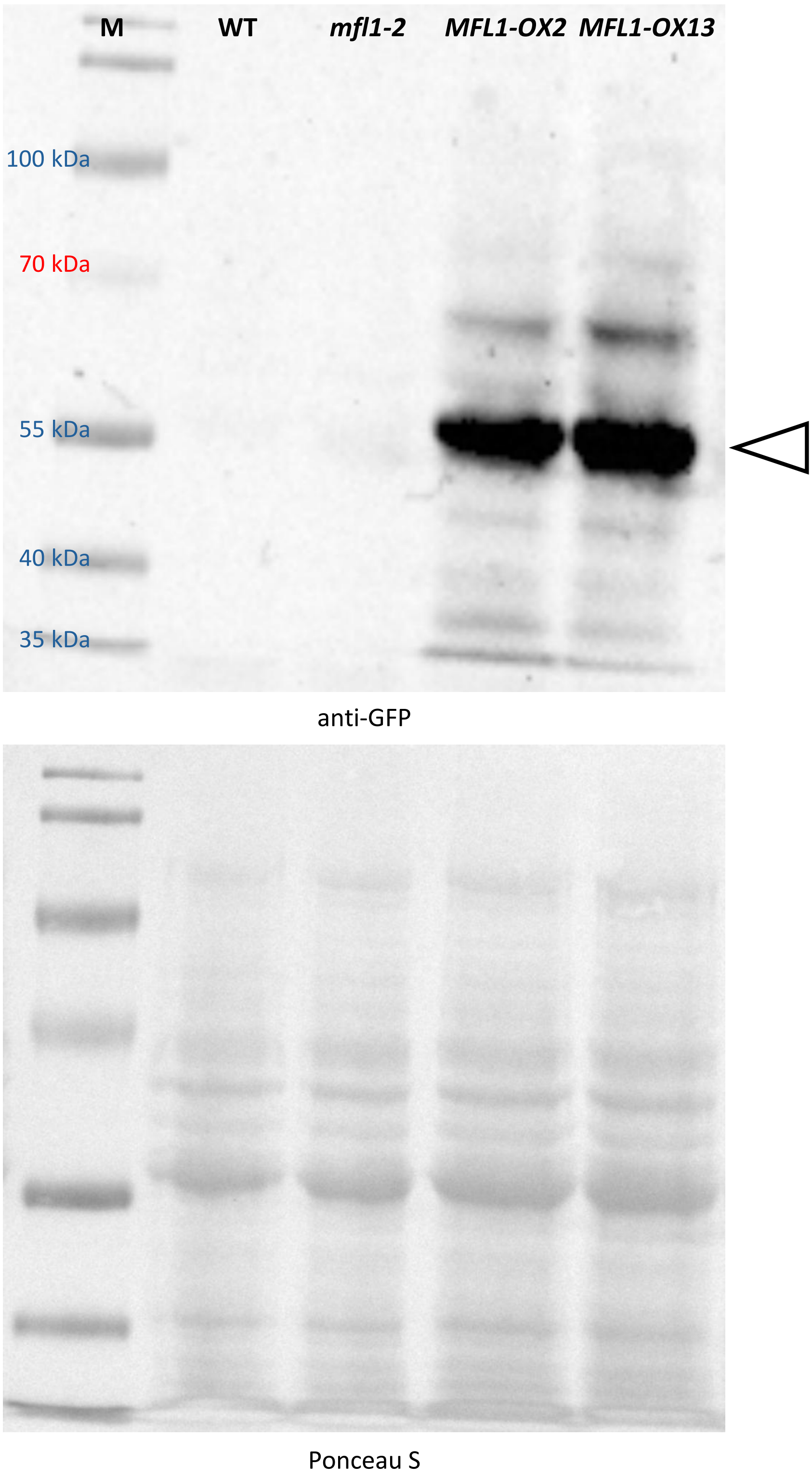


**Figure S2.** Confirmation of AtMFL1 protein expression in chloroplast fractions from leaves of *A. thaliana.*

Western blot analysis of AtMFL1 protein was performed using anti-GFP antibodies and chloroplast fractions obtained from leaves of wild type plants and *atmfl1-2* knock-out mutant, as well as *AtMFL1-OX2* and *AtMFL1-OX13* lines. The presented image is representative of three biological replicates. An arrow indicates the AtMFL1.

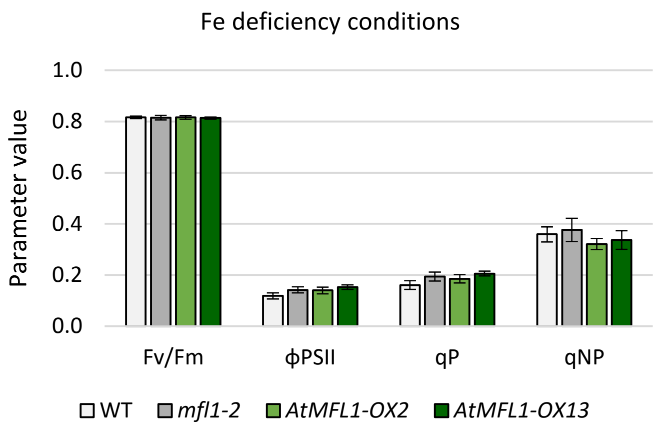


**Figure S3.** Photosynthesis parameters of *atmfl1-2* and *AtMFL1-OX* mutants under iron deficiency conditions.

Photosynthesis parameters were measured in 8-week-old plants under Fe deficiency conditions (medium without $FeSO_4$-EDTA supplemented with 0.05 mM BPS). Treatment was applied for two weeks; Fv/Fm - maximum quantum yield of PSII, ϕPSII - quantum yield of PSII, qP - photochemical fluorescence quenching, qNP - nonphotochemical quenching. Presented results are the means of three biological replicates. Error bars represent standard error (±SE). Asterics represent statistically significant differences between wild type and knock-out or overexpression mutants ($p<0.05$; Student *t*-test).

| Fe concentration [mg/kg DW] | | | |
|---|---|---|---|
| control - roots | | | |
| plant line | mean | SE | significance |
| WT | 5983.04 | 598.12 | |
| *mfl1-2* | 5608.92 | 138.77 | ns |
| *MFL1-OX2* | 6925.69 | 262.19 | ns |
| *MFL1-OX13* | 7825.01 | 166.78 | * |
| Fe deficiency - roots | | | |
| plant line | mean | SE | significance |
| WT | 116.72 | 36.28 | |
| *mfl1-2* | 35.74 | 14.10 | ns |
| *MFL1-OX2* | 256.14 | 18.62 | * |
| *MFL1-OX13* | 72.81 | 22.66 | ns |
| Fe excess (0.2 mM) - roots | | | |
| plant line | mean | SE | significance |
| WT | 12011.02 | 812.68 | |
| *mfl1-2* | 10311.95 | 987.00 | ns |
| *MFL1-OX2* | 12215.42 | 595.68 | ns |
| *MFL1-OX13* | 15414.79 | 932.12 | ns |
| control - leaves | | | |
| plant line | mean | SE | significance |
| WT | 102.83 | 5.41 | |
| *mfl1-2* | 153.72 | 9.74 | * |
| *MFL1-OX2* | 97.02 | 11.41 | ns |
| *MFL1-OX13* | 129.36 | 5.11 | * |
| Fe deficiency - leaves | | | |
| plant line | mean | SE | significance |
| WT | 92.10 | 4.64 | |
| *mfl1-2* | 95.79 | 2.31 | ns |
| *MFL1-OX2* | 114.81 | 2.33 | * |
| *MFL1-OX13* | 92.94 | 1.02 | ns |
| Fe excess (0.2 mM) - leaves | | | |
| plant line | mean | SE | significance |
| WT | 131.88 | 26.77 | |
| *mfl1-2* | 79.11 | 0.65 | ns |
| *MFL1-OX2* | 111.91 | 17.18 | ns |
| *MFL1-OX13* | 96.36 | 5.20 | ns |

| Mn concentration [mg/kg DW] | | | |
|---|---|---|---|
| control - roots | | | |
| plant line | mean | SE | significance |
| WT | 441.63 | 15.61 | |
| *mfl1-2* | 566.32 | 11.53 | * |
| *MFL1-OX2* | 559.89 | 39.57 | * |
| *MFL1-OX13* | 260.41 | 19.73 | * |
| Fe deficiency - roots | | | |
| plant line | mean | SE | significance |
| WT | 589.34 | 14.62 | |
| *mfl1-2* | 567.09 | 67.51 | ns |
| *MFL1-OX2* | 517.61 | 19.31 | * |
| *MFL1-OX13* | 632.42 | 77.87 | ns |
| Fe excess (0.2 mM) - roots | | | |
| plant line | mean | SE | significance |
| WT | 75.18 | 6.50 | |
| *mfl1-2* | 62.90 | 7.19 | ns |
| *MFL1-OX2* | 62.76 | 1.79 | ns |
| *MFL1-OX13* | 68.73 | 1.24 | ns |
| control - leaves | | | |
| plant line | mean | SE | significance |
| WT | 201.16 | 11.42 | |
| *mfl1-2* | 215.51 | 6.01 | ns |
| *MFL1-OX2* | 236.43 | 5.63 | ns |
| *MFL1-OX13* | 232.95 | 3.79 | ns |
| Fe deficiency - leaves | | | |
| plant line | mean | SE | significance |
| WT | 191.57 | 8.15 | |
| *mfl1-2* | 205.52 | 15.31 | ns |
| *MFL1-OX2* | 201.00 | 8.77 | ns |
| *MFL1-OX13* | 223.59 | 16.20 | ns |
| Fe excess (0.2 mM) - leaves | | | |
| plant line | mean | SE | significance |
| WT | 140.77 | 3.37 | |
| *mfl1-2* | 146.95 | 14.39 | ns |
| *MFL1-OX2* | 149.01 | 2.31 | ns |
| *MFL1-OX13* | 141.96 | 1.67 | ns |

**Table S1.** Accumulation of Fe and Mn in roots and leaves of *A. thaliana.*

Fe and Mn levels were measured in roots and leaves of 8-week-old wild-type, *atmfl1-2* mutant, and two *AtMFL1* overexpressing lines grown under control conditions (control), Fe deficiency (medium without $FeSO_4$-EDTA with added 0.05 mM BPS for two weeks), and Fe excess (medium supplemented with $FeSO_4$-EDTA to a 0.2 mM final concentration for two weeks). All presented values are the means of three separate biological replicates. Significance was assayed by Student *t*-test (*<0.05). Asterics represent statistically significant differences between wild type and knock-out or overexpression mutants. DW - dry weight, SE - standard error.

| Zn concentration [mg/kg DW] | | | |
|---|---|---|---|
| control - roots | | | |
| plant line | mean | SE | significance |
| WT | 552.71 | 16.48 | |
| *mfl1-2* | 649.11 | 9.16 | * |
| *MFL1-OX2* | 853.10 | 32.23 | * |
| *MFL1-OX13* | 1037.57 | 4.12 | * |
| Fe deficiency - roots | | | |
| plant line | mean | SE | significance |
| WT | 1177.63 | 128.11 | |
| *mfl1-2* | 1215.04 | 27.87 | ns |
| *MFL1-OX2* | 1457.08 | 162.67 | ns |
| *MFL1-OX13* | 2060.16 | 159.31 | * |
| Fe excess (0.2 mM) - roots | | | |
| plant line | mean | SE | significance |
| WT | 393.14 | 19.63 | |
| *mfl1-2* | 404.19 | 67.54 | ns |
| *MFL1-OX2* | 414.58 | 1.52 | ns |
| *MFL1-OX13* | 539.48 | 31.41 | * |
| control - leaves | | | |
| plant line | mean | SE | significance |
| WT | 166.75 | 10.83 | |
| *mfl1-2* | 180.53 | 7.87 | ns |
| *MFL1-OX2* | 195.96 | 4.93 | ns |
| *MFL1-OX13* | 267.01 | 7.19 | * |
| Fe deficiency - leaves | | | |
| plant line | mean | SE | significance |
| WT | 170.48 | 11.90 | |
| *mfl1-2* | 172.86 | 6.12 | ns |
| *MFL1-OX2* | 193.37 | 9.15 | ns |
| *MFL1-OX13* | 218.54 | 14.46 | ns |
| Fe excess (0.2 mM) - leaves | | | |
| plant line | mean | SE | significance |
| WT | 123.56 | 5.10 | |
| *mfl1-2* | 132.88 | 4.49 | ns |
| *MFL1-OX2* | 126.67 | 6.83 | ns |
| *MFL1-OX13* | 127.81 | 4.04 | ns |

| Cu concentration [mg/kg DW] | | | |
|---|---|---|---|
| control - roots | | | |
| plant line | mean | SE | significance |
| WT | 62.59 | 3.58 | |
| *mfl1-2* | 64.28 | 3.58 | ns |
| *MFL1-OX2* | 63.54 | 7.66 | ns |
| *MFL1-OX13* | 41.37 | 0.81 | * |
| Fe deficiency - roots | | | |
| plant line | mean | SE | significance |
| WT | 74.48 | 5.56 | |
| *mfl1-2* | 91.24 | 6.86 | ns |
| *MFL1-OX2* | 84.43 | 10.18 | ns |
| *MFL1-OX13* | 85.15 | 2.78 | ns |
| Fe excess (0.2 mM) - roots | | | |
| plant line | mean | SE | significance |
| WT | 55.32 | 3.45 | |
| *mfl1-2* | 50.87 | 5.90 | ns |
| *MFL1-OX2* | 44.19 | 2.08 | ns |
| *MFL1-OX13* | 36.30 | 0.49 | * |
| control - leaves | | | |
| plant line | mean | SE | significance |
| WT | 3.53 | 0.17 | |
| *mfl1-2* | 4.22 | 0.38 | ns |
| *MFL1-OX2* | 6.07 | 0.13 | * |
| *MFL1-OX13* | 6.21 | 0.43 | * |
| Fe deficiency - leaves | | | |
| plant line | mean | SE | significance |
| WT | 7.82 | 0.38 | |
| *mfl1-2* | 11.62 | 0.08 | * |
| *MFL1-OX2* | 9.47 | 1.62 | ns |
| *MFL1-OX13* | 8.99 | 1.94 | ns |
| Fe excess (0.2 mM) - leaves | | | |
| plant line | mean | SE | significance |
| WT | 4.37 | 0.50 | |
| *mfl1-2* | 8.44 | 0.14 | * |
| *MFL1-OX2* | 6.44 | 0.76 | ns |
| *MFL1-OX13* | 5.03 | 0.46 | ns |

**Table S2.** Accumulation of Zn and Cu in roots and leaves of *A. thaliana.*

Zn and Cu levels were measured in roots and in leaves of 8-week-old wild-type, *atmfl1-2,* and two separate *AtMFL1* overexpressing lines under control conditions (control), Fe deficiency (medium without $FeSO_4$-EDTA with added 0.05 mM BPS for two weeks), and Fe excess (medium supplemented with $FeSO_4$-EDTA to a 0.2 mM final concentration for two weeks). All presented values are the means of three separate biological replicates. Significance was assayed by Student *t*-test (*<0.05). Asterics represent statistically significant differences between wild type and knock-out or overexpression mutants. DW - dry weight, SE - standard error.

| roots - control | | | | roots - Fe deficiency | | | | roots - Fe excess (0.2 mM) | | | |
|---|---|---|---|---|---|---|---|---|---|---|---|
| IRT1 | | | | | | | | | | | |
| plant line | mean | SE | significance | plant line | mean | SE | significance | | | | |
| WT | 1 | 0.051049 | | WT | 28.85018 | 2.742304 | | | | | |
| *mfl1-2* | 0.295006 | 0.048729 | * | *mfl1-2* | 17.92543 | 1.073205 | * | | | | |
| *MFL1-OX2* | 0.395342 | 0.016337 | * | MFL1-OX2 | 19.46883 | 1.546301 | * | | | | |
| *MFL1-OX13* | 0.289917 | 0.032475 | * | MFL1-OX13 | 11.07153 | 0.143059 | * | | | | |
| FRO2 | | | | | | | | | | | |
| plant line | mean | SE | significance | plant line | mean | SE | significance | | | | |
| WT | 1 | 0.016364 | | WT | 13.87294 | 0.383963 | | | | | |
| *mfl1-2* | 0.536669 | 0.017644 | * | *mfl1-2* | 8.606907 | 0.398684 | * | | | | |
| *MFL1-OX2* | 0.901426 | 0.016207 | * | *MFL1-OX2* | 9.478867 | 0.398496 | * | | | | |
| *MFL1-OX13* | 0.440174 | 0.022569 | * | *MFL1-OX13* | 6.123409 | 0.060514 | * | | | | |
| FRO3 | | | | | | | | | | | |
| plant line | mean | SE | significance | plant line | mean | SE | significance | plant line | mean | SE | significance |
| WT | 1 | 0.02444 | | WT | 9.333448 | 0.343969 | | WT | 0.749742 | 0.058626 | |
| *mfl1-2* | 0.880940 | 0.023749 | * | *mfl1-2* | 4.807135 | 0.150293 | * | *mfl1-2* | 0.734043 | 0.070857 | ns |
| *MFL1-OX2* | 0.886173 | 0.062142 | ns | *MFL1-OX2* | 4.864134 | 0.254100 | * | *MFL1-OX2* | 0.665422 | 0.059207 | ns |
| *MFL1-OX13* | 0.925385 | 0.031074 | ns | *MFL1-OX13* | 4.96241 | 0.089734 | * | *MFL1-OX13* | 0.658420 | 0.036560 | ns |
| FER1 | | | | | | | | | | | |
| plant line | mean | SE | significance | plant line | mean | SE | significance | plant line | mean | SE | significance |
| WT | 1 | 0.021288 | | WT | 0.138379 | 0.005575 | | WT | 1.267428 | 0.065543 | |
| *mfl1-2* | 0.459621 | 0.128382 | * | *mfl1-2* | 0.615067 | 0.037994 | * | *mfl1-2* | 1.154277 | 0.053766 | ns |
| *MFL1-OX2* | 0.798701 | 0.030692 | * | *MFL1-OX2* | 0.454098 | 0.046564 | * | *MFL1-OX2* | 0.527541 | 0.013249 | * |
| *MFL1-OX13* | 1.942678 | 0.042314 | * | *MFL1-OX13* | 0.133552 | 0.013942 | ns | *MFL1-OX13* | 1.518660 | 0.239822 | ns |
| FER3 | | | | | | | | | | | |
| plant line | mean | SE | significance | plant line | mean | SE | significance | plant line | mean | SE | significance |
| WT | 1 | 0.435808 | | WT | 0.087832 | 0.008527 | | WT | 3.659636 | 0.753129 | |
| *mfl1-2* | 0.308052 | 0.005333 | ns | *mfl1-2* | 0.111168 | 0.004814 | ns | *mfl1-2* | 1.414604 | 0.066539 | * |
| *MFL1-OX2* | 0.231696 | 0.017245 | ns | *MFL1-OX2* | 0.111980 | 0.020528 | ns | *MFL1-OX2* | 0.334851 | 0.065754 | * |
| *MFL1-OX13* | 0.704513 | 0.007966 | ns | *MFL1-OX13* | 0.038253 | 0.002109 | * | *MFL1-OX13* | 1.700346 | 0.346338 | ns |

**Table S3.** Expression of genes involved in iron homeostasis in roots of *A. thaliana*.

Gene expression analysis of *AtIRT1, AtFRO2, AtFRO3, AtFER1,* and *AtFER3* in roots of 8-week-old wild-type, *atmfl1-2* mutant, and two *AtMFL1* overexpressing lines under control conditions (control), Fe deficiency (−Fe; −$FeSO_4$–EDTA, +0.05 mM BPS for two weeks), and Fe excess (+Fe; +0.2 mM $FeSO_4$–EDTA for two weeks). The obtained values were calculated relative to the reference gene *AtCACS*, according to the ΔΔCT method. Presented results are the means of three biological replicates. Significance was assayed by Student *t*-test (*<0.05). Asterics represent statistically significant differences between wild type and knock-out or overexpression mutants. SE - standard error.

| leaves - control | | | | leaves - Fe deficiency | | | | leaves - Fe excess (0.2 mM) | | | |
|---|---|---|---|---|---|---|---|---|---|---|---|
| FRO3 | | | | | | | | | | | |
| plant line | mean | SE | significance | plant line | mean | SE | significance | plant line | mean | SE | significance |
| WT | 1 | 0.028439 | | WT | 4.224341 | 0.223703 | | WT | 0.960452 | 0.010856 | |
| *mfl1-2* | 0.688515 | 0.039659 | * | *mfl1-2* | 2.899600 | 0.136577 | * | *mfl1-2* | 0.776836 | 0.042830 | * |
| *MFL1-OX2* | 0.677613 | 0.013288 | * | *MFL1-OX2* | 4.334393 | 0.386263 | ns | *MFL1-OX2* | 1.114672 | 0.081263 | ns |
| *MFL1-OX13* | 0.810734 | 0.015862 | * | *MFL1-OX13* | 3.855344 | 0.352246 | ns | *MFL1-OX13* | 0.712835 | 0.039533 | * |
| FRO6 | | | | | | | | | | | |
| plant line | mean | SE | significance | plant line | mean | SE | significance | plant line | mean | SE | significance |
| WT | 1 | 0.057609 | | WT | 1.656808 | 0.098570 | | WT | 1.250267 | 0.025641 | |
| *mfl1-2* | 1.257811 | 0.024298 | * | *mfl1-2* | 1.521231 | 0.019414 | ns | *mfl1-2* | 0.939511 | 0.009400 | * |
| *MFL1-OX2* | 1.194018 | 0.061271 | ns | *MFL1-OX2* | 1.204513 | 0.024201 | * | *MFL1-OX2* | 1.895313 | 0.020437 | * |
| *MFL1-OX13* | 0.969322 | 0.051108 | ns | *MFL1-OX13* | 0.958806 | 0.035289 | * | *MFL1-OX13* | 1.051175 | 0.040769 | * |
| FRO7 | | | | | | | | | | | |
| plant line | mean | SE | significance | plant line | mean | SE | significance | plant line | mean | SE | significance |
| WT | 1 | 0.027536 | | WT | 1.529562 | 0.071453 | | WT | 0.806236 | 0.037527 | |
| *mfl1-2* | 0.949443 | 0.032805 | ns | *mfl1-2* | 0.864144 | 0.056987 | * | *mfl1-2* | 0.741796 | 0.007102 | ns |
| *MFL1-OX2* | 0.841333 | 0.034012 | * | *MFL1-OX2* | 1.023889 | 0.107318 | * | *MFL1-OX2* | 1.200695 | 0.012349 | * |
| *MFL1-OX13* | 0.915975 | 0.044456 | ns | *MFL1-OX13* | 0.826436 | 0.021864 | * | *MFL1-OX13* | 0.64024 | 0.019727 | * |
| FRO8 | | | | | | | | | | | |
| plant line | mean | SE | significance | plant line | mean | SE | significance | plant line | mean | SE | significance |
| WT | 1 | 0.020979 | | WT | 1.060990 | 0.046189 | | WT | 1.161807 | 0.039490 | |
| *mfl1-2* | 0.990082 | 0.003833 | ns | *mfl1-2* | 0.852695 | 0.027043 | * | *mfl1-2* | 1.155236 | 0.251493 | ns |
| *MFL1-OX2* | 0.623028 | 0.033529 | * | *MFL1-OX2* | 0.992350 | 0.122206 | ns | *MFL1-OX2* | 0.954990 | 0.092353 | ns |
| *MFL1-OX13* | 0.989535 | 0.024354 | ns | *MFL1-OX13* | 0.833895 | 0.017955 | * | *MFL1-OX13* | 0.885815 | 0.027971 | * |
| FPN3 | | | | | | | | | | | |
| plant line | mean | SE | significance | plant line | mean | SE | significance | plant line | mean | SE | significance |
| WT | 1 | 0.066969 | | WT | 1.305571 | 0.044283 | | WT | 0.770465 | 0.020354 | |
| *mfl1-2* | 0.895893 | 0.018252 | ns | *mfl1-2* | 1.406446 | 0.029702 | ns | *mfl1-2* | 0.831574 | 0.026008 | ns |
| *MFL1-OX2* | 1.138120 | 0.022290 | ns | *MFL1-OX2* | 1.342727 | 0.074762 | ns | *MFL1-OX2* | 1.184612 | 0.075605 | * |
| *MFL1-OX13* | 0.913292 | 0.051808 | ns | *MFL1-OX13* | 0.920184 | 0.018191 | * | *MFL1-OX13* | 0.859027 | 0.047448 | ns |
| PIC1 | | | | | | | | | | | |
| plant line | mean | SE | significance | plant line | mean | SE | significance | plant line | mean | SE | significance |
| WT | 1 | 0.044543 | | WT | 1.513112 | 0.065132 | | WT | 1.454448 | 0.057999 | |
| *mfl1-2* | 0.909559 | 0.053277 | ns | *mfl1-2* | 1.432962 | 0.069539 | ns | *mfl1-2* | 2.193853 | 0.771721 | ns |
| *MFL1-OX2* | 0.678790 | 0.391747 | ns | *MFL1-OX2* | 0.964162 | 0.035803 | * | *MFL1-OX2* | 1.140702 | 0.026128 | * |
| *MFL1-OX13* | 0.781757 | 0.034744 | * | *MFL1-OX13* | 0.805865 | 0.028378 | * | *MFL1-OX13* | 1.147328 | 0.074822 | * |
| FER1 | | | | | | | | | | | |
| plant line | mean | SE | significance | plant line | mean | SE | significance | plant line | mean | SE | significance |
| WT | 1 | 0.058483 | | WT | 0.089407 | 0.004787 | | WT | 3.911469 | 0.404506 | |
| *mfl1-2* | 0.585326 | 0.113397 | * | *mfl1-2* | 0.388198 | 0.021750 | * | *mfl1-2* | 1.684403 | 0.100956 | * |
| *MFL1-OX2* | 1.780563 | 0.144210 | * | *MFL1-OX2* | 0.131752 | 0.002676 | * | *MFL1-OX2* | 1.919354 | 0.152753 | * |
| *MFL1-OX13* | 0.736169 | 0.033049 | * | *MFL1-OX13* | 0.117505 | 0.007398 | * | *MFL1-OX13* | 1.697865 | 0.116954 | * |
| FER3 | | | | | | | | | | | |
| plant line | mean | SE | significance | plant line | mean | SE | significance | plant line | mean | SE | significance |
| WT | 1 | 0.104127 | | WT | 0.215098 | 0.018982 | | WT | 2.893278 | 0.099983 | |
| *mfl1-2* | 0.888521 | 0.059179 | ns | *mfl1-2* | 0.413857 | 0.039012 | * | *mfl1-2* | 2.047880 | 0.112101 | * |
| *MFL1-OX2* | 1.477353 | 0.031745 | * | *MFL1-OX2* | 0.269286 | 0.036141 | ns | *MFL1-OX2* | 1.970321 | 0.114225 | * |
| *MFL1-OX13* | 1.371872 | 0.050654 | * | *MFL1-OX13* | 0.199173 | 0.006498 | ns | *MFL1-OX13* | 1.298345 | 0.140510 | * |
| FER4 | | | | | | | | | | | |
| plant line | mean | SE | significance | plant line | mean | SE | significance | plant line | mean | SE | significance |
| WT | 1 | 0.031775 | | WT | 0.165548 | 0.005333 | | WT | 1.462446 | 0.051011 | |
| *mfl1-2* | 0.886611 | 0.047223 | * | *mfl1-2* | 0.387654 | 0.025207 | * | *mfl1-2* | 0.840348 | 0.006558 | * |
| *MFL1-OX2* | 1.671036 | 0.029594 | * | *MFL1-OX2* | 0.217598 | 0.013028 | * | *MFL1-OX2* | 1.230044 | 0.054631 | * |
| *MFL1-OX13* | 1.021771 | 0.049049 | * | *MFL1-OX13* | 0.165865 | 0.011536 | ns | *MFL1-OX13* | 1.418269 | 0.093399 | ns |

**Table S4.** Expression of genes involved in iron homeostasis in leaves of *A. thaliana.*

Gene expression analysis of *AtFRO3, AtFRO6, AtFRO7, AtFRO8, AtFPN3, AtPIC1, AtFER1, AtFER3,* and *AtFER4* in leaves of 8-week-old wild type, *atmfl1-2* mutant, and two *AtMFL1* overexpressing lines under control conditions (control), Fe deficiency (−Fe; −$FeSO_4$–EDTA, +0.05 mM BPS for two weeks), and Fe excess (+Fe; +0.2 mM $FeSO_4$–EDTA for two weeks). The obtained values were calculated relative to the reference gene *AtCACS,* according to the ΔΔCT method. Presented results are the means of three biological replicates. Significance was assayed by Student *t*-test (*<0.05). Asterics represent statistically significant differences between wild type and knock-out or overexpression mutants. SE - standard error.

| primer name | sequence | source |
|---|---|---|
| primers used for cloning into pUG23 vector | | |
| AtMFL1 for.23 | AAAACTAGTATGGAAGCTAGACTCTCCG | this study |
| AtMFL1 rev.23 | TTTGTCGACAGAATCAGCAGCAACGTTAG | this study |
| CsMFL1 for.23 | AAAACTAGTATGGAAACCAGGCTCTCCG | this study |
| CsMFL1 rev.23 | TTTGTCGACAGTTGAAGCAGAAGCCAATTCTG | this study |
| primers used for cloning into pA7 vector | | |
| AtMFL1 for.A7 | AAAGTCGACATGGAAGCTAGACTCTCC | this study |
| AtMFL1 rev.A7 | TTTACTAGTAGAATCAGCAGCAACGTTAGC | this study |
| AtMFL1-92 for.A7 | AAAGTCGACATGCCCGCATCACGAAG | this study |
| CsMFL1 for.A7 | AAAGTCGACATGGAAACCAGGCTCTCCG | this study |
| CsMFL1 rev.A7 | TTTACTAGTAGTTGAAGCAGAAGCCAATTCTG | this study |
| CsMFL1-79 for.A7 | AAAGTCGACATGCCAAAAATCCAATCCCT | this study |
| primers used for cloning into pDONR vector | | |
| AtMFL1 for.DONR | GGACAAGTTTGTACAAAAAAGCAGGCTTAATGGCAACAGAAGCAACAACCA | this study |
| AtMFL1 rev.DONR | GGACCACTTTGTACAAGAAAGCTGGGTCAGCTGCGTTTGCTTCAC | this study |
| primers used for real-time PCR | | |
| AtMFL1 for | GCTCATTACACAGAGAATGCAA | this study |
| AtMFL1 rev | CGACGAATAACTGAGTACACC | this study |
| CsMFL1 for | TTTAGAGAAAGATGGTATAATGGGTC | this study |
| CsMFL1 rev | CTCTGAATTGGTTCAAGTTTGTC | this study |
| AtCACS for | TCAGGAAGGTGTACGGTCA | [82] |
| AtCACS rev | AGGTTTACACTTTCCACAATA | |
| CsCACSfor | TGGGAAGATTCTTATGAAGTGC | [83] |
| CsCACSrev | CTCGTCAAATTTACACATTGGT | |
| AtIRT1 for | GCCCCGCAAATGATGTTACC | [96] |
| AtIRT1 rev | TCCAATGACCACCGAGTGAA | |
| AtFRO2 for | ATCGAAAGTCGCCACACCAT | [96] |
| AtFRO2 rev | GAGCCACAAACATCGCCAAG | |
| AtFRO3 for | GATTCTACTGGCTTCTCTTGG | [58] |
| AtFRO3 rev | CTAATCCGGCCTTCACTAAC | |
| AtFRO6 for | AATCCGAGCCTCGCTTGGA | [97] |
| AtFRO6 rev | TGGTCCGTGGTAGCTTGACAGAA | |
| AtFRO7 for | CACTCTCTTTGGCCTCACAG | [58] |
| AtFRO7 rev | TTAGTGAAAACCGTCTCTTCCC | |
| AtFRO8 for | CGTGTCTCTAACGACTTCAAGA | [58] |
| AtFRO8 rev | CGGCTAATAGACCAAACCTTG | |
| AtFPN3 for | GTGGGTTCTTTGCCAACCATGAC | [60] |
| AtFPN3 rev | TTAGGACGGTCCAGAACTCCAG | |
| AtPIC1 for | GGCTTTAACAACTTCAGCAAAC | [61] |
| AtPIC1 rev | GCAACCTTAGGAACTACGAC | |
| AtFER1 for | TCGTTGAGAGTGAATTTCTGG | [98] |
| AtFER1 rev | ACCCCAACATTGGTCATCTG | |
| AtFER3 for | AGAGTGTGTTTCTGAACGAAC | [98] |
| AtFER3 rev | CCAAACTGCGAGATTACAGC | |
| AtFER4 for | AGAGCGAGTTTCTGACAGAG | [98] |
| AtFER4 rev | CACAGTAGACACAAGAGTCC | |
| primers used for identification of knock-out lines from NASC | | |
| Lba1.3 | ATTTTGCCGATTTCGGAAC | T-DNA primer design |
| mfl1-2 for | AACATGGTGGTCAGAGAATCG | T-DNA primer design |
| mfl1-2 rev | TTGATCCATTTCAGGAATTCG | T-DNA primer design |

**Table S5.** List of primers used in this study.

**References** (not included in the main text):


96. Li, X.; Zhang, H.; Ai, Q.; Liang, G.; Yu, D. Two BHLH Transcription Factors, BHLH34 and BHLH104, Regulate Iron Homeostasis in Arabidopsis Thaliana. *Plant Physiol.* **2016**, *170*, 2478–2493. https://doi.org/10.1104/pp.15.01827.
97. Feng, H.; An, F.; Zhang, S.; Ji, Z.; Ling, H.-Q.; Zuo, J. Light-Regulated, Tissue-Specific, and Cell Differentiation-Specific Expression of the Arabidopsis Fe(III)-Chelate Reductase Gene AtFRO6. *Plant Physiol.* **2006**, *140*, 1345–1354. https://doi.org/10.1104/pp.105.074138.
98. Duc, C.; Cellier, F.; Lobréaux, S.; Briat, J.-F.; Gaymard, F. Regulation of Iron Homeostasis in Arabidopsis Thaliana by the Clock Regulator Time for Coffee. *J. Biol. Chem.* **2009**, *284*, 36271–36281. https://doi.org/10.1074/jbc.M109.059873.
